# C-reactive protein and D-dimer in cerebral vein thrombosis: Relation to clinical and imaging characteristics as well as outcomes in a French cohort study

**DOI:** 10.1016/j.rpth.2023.100130

**Published:** 2023-03-28

**Authors:** Paul Billoir, Virginie Siguret, Elisabeth Masson Fron, Ludovic Drouet, Isabelle Crassard, Raphaël Marlu, Marianne Barbieux-Guillot, Pierre-Emmanuel Morange, Emmanuelle Robinet, Catherine Metzger, Valérie Wolff, Elisabeth André-Kerneis, Frédéric Klapczynski, Brigitte Martin-Bastenaire, Fernando Pico, Fanny Menard, Emmanuel Ellie, Geneviève Freyburger, François Rouanet, Hong-An Allano, Gaëlle Godenèche, Guillaume Mourey, Thierry Moulin, Micheline Berruyer, Laurent Derex, Catherine Trichet, Gwénaëlle Runavot, Agnès Le Querrec, Fausto Viader, Sophie Cluet-Dennetiere, Thomas Tarek Husein, Magali Donnard, Francisco Macian-Montoro, Catherine Ternisien, Benoît Guillon, Sophie Laplanche, Mathieu Zuber, Jean-Yves Peltier, Philippe Tassan, Bertrand Roussel, Sandrine Canaple, Emilie Scavazza, Nicolas Gaillard, Aude Triquenot Bagan, Véronique Le Cam Duchez

**Affiliations:** 1 Univ Rouen Normandie, INSERM U1096, Department of Vascular Hemostasis Unit, CHU Rouen, Rouen, France; 2 Department of Biological Hematology, Lariboisière University Hospital, Université Paris Cité, Paris, France; 3 Department of Neurology, Lariboisière University Hospital, Université Paris Cité, Paris, France; 4 Department of Biological Hematology, Grenoble University Hospital, Grenoble, France; 5 Department of Neurology, Grenoble University Hospital, Grenoble, France; 6 Department of Biological Hematology, Marseille University Hospital, Marseille, France; 7 Department of Neurology, Marseille University Hospital, Marseille, France; 8 Department of Biological Hematology, Strasbourg University Hospital, Strasbourg, France; 9 Department of Neurology, Strasbourg University Hospital, Strasbourg, France; 10 Department of Biological Hematology, Meaux Hospital, Meaux, France; 11 Department of Neurology, Meaux Hospital, Meaux, France; 12 Department of Biological Hematology, Versailles Hospital, Versailles, France; 13 Department of Neurology, Versailles Hospital, Versailles, France; 14 Department of Biological Hematology, Bayonne Hospital, Bayonne, France; 15 Department of Neurology, Bayonne Hospital, Bayonne, France; 16 Department of Biological Hematology, Bordeaux University Hospital, Bordeaux, France; 17 Department of Neurology, Bordeaux University Hospital, Bordeaux, France; 18 Department of Biological Hematology, La Rochelle Hospital, La Rochelle, France; 19 Department of Neurology, La Rochelle Hospital, La Rochelle, France; 20 Department of Biological Hematology, Besançon University Hospital, Besançon, France; 21 Department of Neurology, Besançon University Hospital, Besançon, France; 22 Department of Biological Hematology, Lyon University Hospital, Lyon, France; 23 Department of Neurology, Lyon University Hospital, Lyon, France; 24 Department of Biological Hematology, Argenteuil Hospital, Argenteuil, France; 25 Department of Neurology, Argenteuil Hospital, Argenteuil, France; 26 Department of Biological Hematology, Caen University Hospital, Caen, France; 27 Department of Neurology, Caen University Hospital, Caen, France; 28 Department of Biological Hematology, Compiègne Hospital, Compiègne, France; 29 Department of Neurology, Compiègne Hospital, Compiègne, France; 30 Department of Biological Hematology, Limoges University Hospital, Limoges, France; 31 Department of Neurology, Limoges University Hospital, Limoges, France; 32 Department of Biological Hematology, Nantes University Hospital, Nantes, France; 33 Department of Neurology, Nantes University Hospital, Nantes, France; 34 Department of Biological Hematology, Saint Joseph Hospital, Paris, France; 35 Department of Neurology, Saint Joseph Hospital, Paris, France; 36 Department of Biological Hematology, Poissy Hospital, Poissy, France; 37 Department of Neurology, Poissy Hospital, Poissy, France; 38 Department of Biological Hematology, Amiens university Hospital, Amiens, France; 39 Department of Neurology, Amiens university Hospital, Amiens, France; 40 Department of Biological Hematology, Perpignan Hospital, Perpignan, France; 41 Department of Neurology, Perpignan Hospital, Perpignan, France; 42 Department of Neurology, CHU Rouen, Rouen, France

**Keywords:** cerebral venous sinus thrombosis, D-dimer, high-sensitivity C-reactive protein, thrombin generation assay

## Abstract

**Introduction:**

Cerebral venous sinus thrombosis (CVST) is a rare disease with highly variable clinical presentation and outcomes. Clinical studies suggest a role of inflammation and coagulation in CVST outcomes. The aim of this study was to investigate the association of inflammation and hypercoagulability biomarkers with CVST clinical manifestations and prognosis.

**Methods:**

This prospective multicenter study was conducted from July 2011 to September 2016. Consecutive patients referred to 21 French stroke units and who had a diagnosis of symptomatic CVST were included. High-sensitivity C-reactive protein (hs-CRP), neutrophil-to-lymphocyte ratio (NLR), D-dimer, and thrombin generation using calibrated automated thrombogram system were measured at different time points until 1 month after anticoagulant therapy discontinuation.

**Results:**

Two hundred thirty-one patients were included. Eight patients died, of whom 5 during hospitalization. The day 0 hs-CRP levels, NLR, and D-dimer were higher in patients with initial consciousness disturbance than in those without (hs-CRP: 10.2 mg/L [3.6-25.5] vs 23.7 mg/L [4.8-60.0], respectively; NLR: 3.51 [2.15-5.88] vs 4.78 [3.10-9.59], respectively; D-dimer: 950 μg/L [520-2075] vs 1220 μg/L [950-2445], respectively). Patients with ischemic parenchymal lesions (n = 31) had a higher endogenous thrombin potential_5pM_ than those with hemorrhagic parenchymal lesions (n = 31): 2025 nM min (1646-2441) vs 1629 nM min (1371-2090), respectively (*P* = .0082). Using unadjusted logistic regression with values >75th percentile, day 0 hs-CRP levels of >29.7 mg/L (odds ratio, 10.76 [1.55-140.4]; *P* = .037) and day 5 D-dimer levels of >1060 mg/L (odds ratio, 14.63 [2.28-179.9]; *P* = .010) were associated with death occurrence.

**Conclusion:**

Two widely available biomarkers measured upon admission, especially hs-CRP, could help predict bad prognosis in CVST in addition to patient characteristics. These results need to be validated in other cohorts.

## Essentials

• There are several clinical presentations of cerebral venous sinus thrombosis (CVST).
• Association between inflammation and CVST severity has been observed.
• D-dimer and C-reactive protein predicted bad prognosis in CVST.
• Elevated endogenous thrombin potential on admission was associated with ischemic parenchymal lesions.

## 1 Introduction

Cerebral venous sinus thrombosis (CVST) is a rare thrombotic disorder with an estimated incidence of 1.3 to 1.57 per 100,000 person-years from studies of various European and Australian populations and 2.85 to 3.16 in the United States [[1], [2], [3], [4]]. Several risk factors for CVST have been identified, including oral contraceptive use; pregnancy; thrombophilia; inflammatory diseases; infections; trauma; malignancy; medications, such as steroids; endocrine, and hematologic disorders [1,[3], [4], [5]]. Delays in diagnosis of CVST are common and substantial, with the first symptoms occurring from less than 48 hours until beyond 30 days [5]. Four main patterns have been identified as CVST clinical manifestations: isolated intracranial hypertension, focal syndrome, diffuse encephalopathy, and cavernous sinus syndrome [6]. Clinical presentation of CVST is affected by the patient’s age, the time between the onset of symptoms and the admission to hospital, location of CVST, and the presence of cerebral parenchymal lesions.

Only a few studies have focused on the potential interest of biomarkers in CVST diagnosis and prognosis. Although plasma D-dimer has been widely validated as an effective tool for excluding the diagnosis of deep venous thrombosis or pulmonary embolism, its potential usefulness in the management of patients with suspected CVST is debated [7,8]. Indeed, D-dimer testing is of little use for CVST diagnosis [9]. No study has yet evaluated the association of D-dimer with CVST clinical manifestations. Besides, thrombin generation (TG) has been proposed as a global assay to evidence hypocoagulable [10,11] and hypercoagulable states [[12], [13], [14]]: increased TG is an independent predictor of recurrent venous thrombosis [15]. Inflammation biomarkers, such as fibrinogen and high-sensitivity C-reactive protein (hs-CRP), have been recently described as possible predictors of poor prognosis in CVST [16]. The neutrophil-to-lymphocyte ratio (NLR) reflects subclinical inflammation, predicting mortality in myocardial infarction and cardiovascular risk [17].

We recently described the clinical and imaging features of patients presenting with CVST enrolled in a French prospective cohort [18]. The aim of the present study was to investigate, in the French prospective cohort, the association of inflammation biomarkers, D-dimer, and TG parameters with clinical or imaging characteristics in the patients with CVST in this cohort at diagnosis and during the follow-up months.

## 2 Methods

### 2.1 Population study

The “French prospective cohort–cerebral vein thrombosis” (FPCCVT) multicenter study was prospectively conducted from July 2011 to September 2016 [18]. Briefly, symptomatic patients with CVST from 21 French stroke unit centers were included. This study was funded through a clinical research program (N° 2010/087/HP) of the French Ministry of Health and registered on clinicaltrials.gov (NCT02013635). Patients were included regardless of race/ethnicity, and fully covered by the French health system.

### 2.2 Clinical and imaging characteristics

The diagnosis of CVST had to be confirmed by cerebral tomodensitometry venography and/or magnetic resonance imaging (MRI) combined with magnetic resonance venography and/or digital subtraction angiography following established diagnostic criteria [19]. Patients’ data were recorded. Clinical status was evaluated at admission using the Glasgow Coma Scale and the National Institutes of Health Stroke Scale (NIHSS). Presenting syndromes were categorized as isolated intracranial hypertension and/or isolated headache, focal syndrome, cavernous sinus syndrome, and diffuse encephalopathy. Radiological evolution was evaluated at 3 months using preferably MRI with magnetic resonance venography. “Partial recanalization” was defined as recanalization of at least one previously thrombosed sinus or vein in an individual patient with CVST. “No recanalization” was defined as total absence of any type of recanalization in any previously thrombosed sinus or vein. “Complete recanalization” was considered when all previously thrombosed structures met the criteria of complete recanalization. Three categories were predefined according to the time from the onset of symptoms to diagnosis: within 2 days (acute), between 2 and 30 days (subacute), and beyond 30 days (chronic).

The extent of the parenchymal lesions was classified as minor (≤1/3 of lobe lesions), moderate (between 1/3 and 2/3 of lobe lesions), and severe (>2/3 of lobe lesions). The type of parenchymal lesion was described as ischemic, hemorrhagic, or both. Follow-up visits were planned at 3 months, and one month after stopping an antithrombotic treatment or after a maximum of 13 months if antithrombotic treatment was continued [18].

### 2.3 Laboratory parameters and assays

Laboratory tests, including whole blood count and plasma fibrinogen, were locally performed for each patient upon admission before any initiation of anticoagulant therapy (D_0_). Additional blood was collected on EDTA, 0.109M citrate, and dry tubes to build a plasma and serum bank. In addition to D_0_ sampling, blood samples were taken at different times, especially at day 5 (D_5_) after the diagnosis.

Anemia was defined by decreased hemoglobin <12.0 g/dL in females and 13.0 g/dL in males.

Platelet-poor plasma (PPP) was prepared as recommended by the “Groupe Français d’études sur l’Hémostase et la Thrombose,” ie, double-centrifuged at 2000 g for 10 minutes, and aliquots were kept frozen at −80 °C (www.geht.org). Samples were transported on dry ice for central analysis. Plasma D-dimer was assessed using Liatest DDI+ (Diagnostica Stago) on a STAR analyzer and serum hs-CRP using C-reactive protein (CRP)-Vario on a C8000 analyzer (Abbott) at Lariboisière Hospital (Paris). TG was performed on a calibrated automated thrombogram system using a microplate fluorometer (Fluorocan Ascent, Thermoscientific Labsystems) at Rouen University Hospital. PPP samples were run using either PPP reagent (5pM tissue factor and 4μM phospholipid vesicles) or low PPP reagent (1pM tissue factor and 4μM phospholipid vesicles) (Diagnostica Stago). We recorded and analyzed the following parameters: lag time, time to peak, endogenous thrombin potential (ETP) (area under the thrombin time-concentration curve), maximal thrombin peak, and velocity. TG assay was not performed if patients were on anticoagulant therapy before D_0_ sampling; activity was systematically measured to detect anti-Xa inhibitors.

### 2.4 Statistical analysis

The Shapiro-Wilk test was used to test normality of continuous variables. Normally, distributed data were presented as mean ± standard deviations and non-Gaussian variables as median ± IQR. We performed analyses to test relationships between biomarkers, namely inflammation biomarkers, D-dimer, and TG parameters measured at different times and i/clinical presentation or imaging data upon admission; ii//clinical evolution evaluated by modified Rankin Score, NIHSS, and death occurrence; iii/clinical/imaging evolution on 3 months. Univariate subgroup comparisons were analyzed using the Mann–Whitney U-test. Multiple group comparisons were performed using the Kruskal–Wallis test completed with the two-stage step-up method of Benjamini, Krieger, and Yekutieli. The correlation between clinical and biological parameters was evaluated with Spearman and Pearson correlation. Univariate logistic regression analysis of clinical outcome (death, presence of a parenchymal lesion) was performed using the following variables as predictors: age >75th percentile, sex, body mass index, dyslipidemia, tobacco, diabetes, initial consciousness disturbances, D-dimer >75th percentile, hs-CRP >75th percentile, and maximal thrombin peak. *P* values of <.05 were considered significant. Data were analyzed using GraphPad Prism 9.4 and the R software, version 4.0.0.

## 3 Results

### 3.1 Clinical characteristics

Two hundred thirty-one patients with a diagnosis of CVST were included. The main demographic and clinical characteristics at baseline are shown in Table 1. Briefly, the median age was 41.2 years (IQR, 28-51). The cohort comprised a majority of women (68.4%), 67.1% of whom took oral contraceptives. The median time from the onset of symptoms to diagnosis was 5 days (range, 0-205). The mode of onset was acute in 82 patients, subacute in 131, and chronic in 18. The median number of vein sinuses with thrombosis was 3 (IQR, 1-15). Twenty-nine (12.5%) had only 1 vein with thrombosis. Thirty-one (13.4%) had isolated thrombotic parenchymal lesions, 31 (13.4%) had isolated hemorrhagic parenchymal lesions, and 27 (11.7%) had both. The median duration of follow-up was 11.9 months (range, 76-543 months) for 197 (88.3%) of 223 surviving patients. Five patients died during hospitalization, 4 of brain herniation (despite decompressive craniectomy in 1 patient), and 1 of uncontrolled cancer. Three more patients died because of underlying conditions such as cancer at 3 (n = 2) and 12 months. Twenty-six were lost to follow-up (they did not come to the control visit at 3 and 12 months). Among the 205 survivor patients who had a complete follow-up, 97 (47.3%) were no longer treated with anticoagulants, 85 patients (41.5%) were still on anticoagulants, and 20 (9.8%) were on aspirin. The median duration of antithrombotic treatments was 356 days (IQR, 228-387).

**Table 1:** Epidemiologic, demographic, and clinical characteristics of patients with cerebral venous sinus thrombosis.

Parameters	All (n = 231)
Demographic characteristics	
Age (y)	41.2 (28-51)
Female	158 (68.4%)
Body mass index (kg/m^2^)	25.4 (22.6-28.5)
Underlying comorbidity	
Dyslipidemia	27 (11.7%)
Hypothyroidism	12 (5.2%)
Diabetes	11 (4.8%)
Smoking history	32 (13.9%)
History of thrombosis	36 (15.6%)
Contraceptive	106 (67.1%)
Initial clinical presentation	
Time between onset of symptoms and diagnosis (d)	5 (range, 0-205)
≤2	82
2-30	131
>30	18
Clinical presentation	
Isolated intracranial hypertension	117 (50.6%)
Focal syndrome	96 (41.5%)
Diffuse encephalopathy	16 (6.9%)
Initial consciousness disturbances	35 (15.2%)
Median GCS score	15 (8-15)
Mean NIHSS score	1.59 (range, 0-26)
0	149 (64.5%)
≤5	208 (90%)
>5	23 (10%)
Initial imaging	
Number of sinus thrombosis	
1	29 (12.6%)
2	33 (14.3)
3	58 (25.1)
4	29 (12.6%)
5	26 (11.3)
6	14 (6%)
7	9 (3.9%)
8	8 (3.5%)
≥9	6 (2.6%))
Parenchymal lesion severity	
Minor	57 (24.7%)
Moderate	25 (10.8%)
Severe	4 (1.7%)
Type of lesion	
Ischemic	31 (13.4%)
Hemorrhagic	31 (13.4%)
both	27 (11.7%)
Evolution	
NIHSS score variation between day 0 and 5	
Stable	159 (68.8%)
Improvement	59 (25.5%)
Worsening	11 (4.8%)
Modified Rankin Score variation between day 5 and month 3	
Stable	95 (41.1%)
Improvement	70 (30.3%)
Worsening	47 (20.3%)

GCS, Glasgow Coma Scale; NIHSS, National Institutes of Health Stroke Scale.

### 3.2 Laboratory parameters and clinical presentation characteristics

Laboratory results obtained at D_0_ are shown in Supplementary Table 1. They are displayed according to the time between the onset of symptoms and diagnosis and the different clinical presentations (Table 2).

**Table 2:** Laboratory markers on D_0_ were associated with the initial clinical presentation.

Biomarkers	Time to diagnosis			Clinical presentation			Initial consciousness	
	<2 d	2-30 d	>30 d	ICHT	Focal syndrome	Diffuse encephalopathy	No initial consciousness disturbance	Initial consciousness disturbance
hs-CRP (mg/L)	10.8 (5.0-29.6)	10.6 (3.2-28.5)	5.3 (2.2-33.4)	8.7 (2.2**-**23.3)**α, β**	13.4 (5.6**-**30.2)**α**	31.0 (13.0**-**66.5)**β**	10.17 (3.6**-**25.5)**γ**	23.71 (4.80**-**60.0)**γ**
NLR	3.40 (2.06-5.42)	3.40 (2.06-5.42)	3.46 (2.29-5.83)	3.32 (2.13-5.87)	4.13 (2.15-6.46)	4.00 (2.15-7.19)	3.51 (2.12**-**5.88)**δ**	4.78 (3.10**-**9.59)**δ**
D-dimer (μg/L)	830 (475-2365)	1160 (680-2220)	655 (413-1535)	925 (500-1680)	1170 (670-2425)	1220 (625-2370)	950 (520**-**2075)**α**	1220 (950**-**2445)**α**
Fibrinogen (g/L)	4.1 (3.6-4.7)	3.8 (3.3-4.6)	4.7 (3.5-5.5)	3.9 (3.4-4.8)	4.3 (3.6-4.7)	3.9 (3.4-4.8)	4.0 (3.4-4.7)	4.3 (3.4-4.8)
Thrombin generation assay 1 pM								
N	82	131	18	106	75	10	148	25
Lagtime_1pM_ (min)	7.9 (5.6-9.2)	7.7 (6.1-8.8)	8.33 (6.9-9.5)	7.5 (5.8-9.0)	7.9 (6.6-8.9)	9.4 (5.9-10.3)	7.8 (6.0-8.9)	8.0 (5.8-10.3)
TTP_1pM_ (min)	11.9 (9.0-13.8)	11.7 (10.1-13.3)	12.3 (9.8-13.2)	11.5 (9.8-13.3)	11.9 (10.2-13.4)	12.9 (8.5-14.8)	11.7 (10.0-13.3)	12.3 (9.1-15.1)
ETP_1pM_ (nM min)	1370 (922-1793)	1314 (1068-1726)	1370 (970-1559)	1333 (929-1699)	1440 (1095-1827)	1141 (998-1463)	1374 (1023-1753)	1121 (954-1505)
Peak_1pM_ (nM)	207 (117-303)	203 (118-269)	209 (101-328)	181 (109-278)	222 (131-295)	218 (127-297)	204 (118-287)	212 (110-271)
Velocity_1pM_ (nM/min)	55.3 (27.0-95.0)	51.6 (24.3-85.5)	63.0 (21.2-131.1)	44.3 (22.1-84.2)	56.2 (29.6-104.1)	70 (31.1-112.0)	54.5 (25.7-86.6)	53.9 (23.9-96.2)
Thrombin generation assay 5 pM								
Lagtime_5pM_ (min)	4.1 (3.3-5.0)	4.0 (3.3-4.9)	4.2 (3.9-5.0)	3.9 (3.3-4.6)	4.3 (3.5-5.4)	4.1 (3.8-5.2)	4.1 (3.3-4.8)	4.2 (3.8-5.8)
TTP_5pM_ (min)	7.5 (5.8-8.5)	7.1 (6.3-8.5)	7.5 (6.7-7.8)	7.1 (6.0-7.7)	7.5 (6.5-9.0)	7.2 (6.2-8.4)	7.1 (6.3-8.3)	7.4 (6.4-9.7)
ETP_5pM_ (nM min)	1645 (1349-2109)	1739 (1417-2107)	1491 (1205-1959)	1652 (1365-2030)	1695 (1422-2255)	1510 (1364-1736)	1743 (1391-2105)	1551 (1316-1782)
Peak_5pM_ (nM)	283 (198-373)	293 (229-389)	278 (209-353)	283 (222-370)	298 (207-411)	279 (238-361)	298 (218-383)	260 (221-347)
Velocity_5pM_ (nM/min)	92.2 (50.8-146)	97.2 (59.6-145.7)	83.7 (66.4-129.6)	91.5 (58.6-141)	97.1 (55.4-148.3)	96.7 (71.8-137.2)	97.0 (57.6-145.9)	80.0 (60.2-124)

Results are expressed as median (IQR).

Statistical analysis was performed using the Mann–Whitney U-test or Kruskal–Wallis test completed with 2-stage step-up method of Benjamini, Krieger, and Yekutieli for multiple comparisons. α: ICHT vs Focal syndrome *P* value = .039; β: ICTH vs Diffuse encephalopathy *P* value = .0045; γ: No initial consciousness disturbance vs initial consciousness disturbance *P* value = .0086; δ: No initial consciousness disturbance vs initial consciousness disturbance *P* value = .012. N was the number of Thrombin generation assay without anticoagulant treatment at D_0_ sampling.

ETP, endogenous thrombin potential; hs-CRP, high-sensitivity C-reactive protein; ICHT, intracranial hypertension; NLR, neutrophil-to-lymphocyte ratio; TTP, time to peak.

#### 3.2.1 Association with the initial clinical presentation

We analyzed the association of biomarkers with symptoms at presentation categorized as isolated intracranial hypertension and/or isolated headache, focal syndrome, cavernous sinus syndrome, and diffuse encephalopathy. A lower D_0_ hs-CRP was observed in patients with isolated intracranial hypertension (8.7 mg/L [IQR, 2.2-23.3]) and diffuse encephalopathy (31.0 mg/L [13.0-66.5]; *P* = .0045). The presence of initial consciousness disturbance was considered a factor of severity. D_0_ hs-CRP levels were significantly higher in patients with initial consciousness disturbance than in those without (hs-CRP: 10.2 mg/L [3.6-25.5] vs 23.7 mg/L [4.8–60.0], respectively; *P* = .0086).

Concerning the NIHSS score, higher D_0_ hs-CRP was associated with worse initial NIHSS score (>5) compared with NIHSS score of ≤5 (*P* = .0009). No significant differences were observed for NLR, fibrinogen, or TG parameters.

Finally, D_0_ NLR (odds ratio [OR], 1.088 [1.011-1.178]; *P* = .0246) and hs-CRP (OR, 1.013 [1.03-1.025]; *P* = .01) were associated with parenchymal lesions.

### 3.3 Laboratory parameters and imaging at presentation

Laboratory parameters on D_0_ according to imaging data are displayed in Table 3.

**Table 3:** Laboratory markers on day 0 were associated with initial imaging results.

Biomarkers	Cerebral parenchymal lesions		Parenchymal lesion severity			Type of lesion		
	Absence of a lesion	Presence of a lesion	Minor	Moderate	Severe	Ischemic	Hemorrhagic	Both
hs-CRP (mg/L)	9.5 (2.6**-**23.2)**α**	15.3 (5.4**-**31.8)**α**	13.4 (5.7-28.5)	18.3 (4.0-52.6)	68.3 (19.8-134.4)	16.1 (7.8-27.5)	10.8 (5.4-30.3)	19.9 (3.6-55.8)
NLR	3.25 (2.08**-**6.07)**β**	4.32 (2.70**-**6.42)**β**	4.32 (2.7-5.9)	4.3 (3.2-10.8)	5.1 (4.1-9.5)	4.0 (2.7-6.0)	5.4 (2.5-6.8)	4.2 (2.8-7.3)
D-dimer (μg/L)	930 (530-1660)	1240 (645-2423)	1070 (543**-**2175)**γ**	2220 (1108**-**3885)**γ**	1760 (900-2620)	1640 (575-2990)	1430 (640-2425)	1050 (733-2313)
Fibrinogen (g/L)	3.8 (3.3-4.7)	4.3 (3.6-4.7)	4.3 (3.6-4.7)	4.3 (3.6-5.2)	4.2 (3.8-4.6)	4.5 (3.7-4.9)	4.1 (3.6-4.6)	4.4 (3.6-5.2)
Thrombin generation assay 1 pM								
N	123	74	40	18	4	25	26	22
Lagtime_1pM_ (min)	7.8 (5.8-9.0)	7.9 (6.7-9.3)	8.0 (7.3-9.4)	7.5 (5.0-9.1)	7.1 (5.6-10.0)	8.1 (7.3-9.4)	7.8 (6.4-8.7)	7.5 (5.0-9.2)
TTP_1pM_ (min)	11.8 (9.9-13.5)	11.9 (10.2-13.4)	12.3 (10.7-13.7)	11.0 (8.3-13.1)	10.0 (8.3-13.3)	12.3 (10.6-13.8)	11.9 (10.1-13.7)	11.0 (8.3-12.9)
ETP_1pM_ (nM min)	1269 (938-1701)	1442 (1090-1835)	1442 (1092-1836)	1454 (1081-1833)	1835 (1349-2176)	1448 (1180-1854)	1406 (1081-1555)	1280 (913-2042)
Peak_1pM_ (nM)	185 (109-279)	221 (139-304)	222 (136-308)	218 (118-292)	336 (217-392)	228 (145-291)	215 (139-284)	218 (89.7-359)
Velocity_1pM_ (nM/min)	44.4 (22.06-84.2)	57.3 (31.4-110.6)	54.3 (27.8-102.4)	56.0 (31.5-110.6)	121.5 (65.2-146)	55.3 (32.2-84.1)	52.5 (31.9-114.8)	67 (20.0-125.7)
Thrombin generation assay 5 pM								
Lagtime_5pM_ (min)	3.96 (3.3-4.8)	4.17 (3.54-5.00)	4.2 (3.4-5.0)	4.2 (3.5-5.4)	4.6 (3.6-5.8)	3.9 (3.5-4.6)	4.2 (3.8-6.0)	4.2 (3.2-5.6)
TTP_5pM_ (min)	7.1 (6.0-8.5)	7.3 (6.5-8.1)	7.3 (6.6-8)	7.3 (6.0-9.0)	7.1 (5.8-9.4)	7.2 (6.5-7.9)	7.5 (6.8-9.4)	7.3 (6.0-9.1)
ETP_5pM_ (nM min)	1627 (1348-2000)	1716 (1422-2163)	1844 (1424-2155)	1477 (1356-2185)	2373 (1469-2450)	2025 (1646**-**2441)**δ**	1629 (1433**-**1797)**δ**	1663 (1080-2124)
Peak_5pM_ (nM)	278 (206-366)	311 (228-419)	337 (237-425)	269 (191-411)	421 (231-485)	361 (264-428)	268 (196-356)	299 (178-427)
Velocity_5pM_ (nM/min)	89.8 (54.7-139)	101.1 (66.0-150.8)	109.2 (70.0-149.0)	92.1 (50.8-173.9)	168.5 (65.4-214.7)	124 (80.6-150.1)	84.9 (50.0-131.3)	85.0 (51.1-185)

Statistical analysis were Mann-Whitney U test or Kruskal–Wallis test completed with two-stage step-up method of Benjamini, Krieger and Yekutieli for multiple comparisons. α: Absence of lesion vs presence of lesion *P* value = .035; β: Absence of lesion vs presence of lesion *P* value = .0013; γ: Minor lesion vs moderate lesion *P* value = .0058; δ: Ischemic lesion vs hemorrhagic lesion *P* value = .048. N was the number of Thrombin generation assay without anticoagulant treatment at D_0_ sampling.

ETP, endogenous thrombin potential; hs-CRP, high-sensitivity C-reactive protein; ICHT, intracranial hypertension; NLR, neutrophil-to-lymphocyte ratio; TTP, time to peak.

#### 3.3.1 Association with cerebral parenchymal lesions

A significant difference in D-dimer levels was also observed in patients with minor vs moderate-to-severe parenchymal lesions (1070 μg/L [543-2175] vs 2220 [1108-3885], respectively; *P* = .0058). Patients with parenchymal ischemic parenchymal lesions (n = 31) had higher ETP_5pM_ than those with hemorrhagic parenchymal lesions (n = 31): 2025 nM min [1646-2441] vs 1629 nM min [1371-2090], respectively; *P* = .0082).

#### 3.3.2 Association with etiological characteristics

D_0_ TG was increased in women under contraceptives: higher ETP_5pM_, maximal thrombin peak_5pM_, and velocity_5pM_ (1892 nM min [IQR, 1573-2407] vs 1489 nM min [IQR, 1489-1840], *P* = .0042; 354 nM [IQR, 255-419] vs 253 [IQR, 189-345], *P* = .0029; and 127.6 nM/min [IQR, 66.0-160.1] vs 51.2 [IQR, 21.2-82.9], *P* < .001; respectively).

### 3.4 Laboratory parameters and evolution

#### 3.4.1 NIHSS evolution from D0 to D5 and biomarkers

We calculated the NIHSS variation from D_0_ to D_5_ (Supplementary Table 2): 11 patients had a worsening score, 159 patients had a stable score, and 59 patients had an improving score. In patients with worsening scores, the D_0_ hs-CRP and NLR were significantly higher than those with stable or improving scores (32.7 mg/L [18.6-105.1] vs 9.6 mg/L [2.8-23.4] vs 13.4 mg/L [5.7-31.4]; 6.9 [4.7-12.7] vs 3.5 [2.1-5.9] vs 3.9 [2.1-6.5]; *P* = .003 and *P* = .0041, respectively).

#### 3.4.2 Death occurrences during hospitalization and biomarkers

Five patients died during hospitalization. No significant correlations were observed between D0 hs-CRP and D-dimer (Supplementary Table 3). The D0 hs-CRP and D-dimer were significantly higher in patients who died during hospitalization (hs-CRP: 71.2 mg/L [IQR, 9.1-110.8] vs 10.6 mg/L [IQR, 3.6-29.1], *P* = .017; D-dimer: 2405 μg/L [IQR, 2085-10250] vs 965 μg/L [IQR, 550-2123], P = .020; respectively). On D_5_, hs-CRP and D-dimer were also higher (hs-CRP: 223.5 mg/L [IQR, 31.3-393.1] vs 4.3 mg/L [IQR, 1.9-12.7], *P* < .0001; D-dimer: 1160 μg/L [IQR, 1140-10,450] vs 650 μg/L [IQR, 380-1030], P = .016; respectively). No significant differences were observed for NLR or TG parameters.

### 3.5 Clinical characteristics and biomarkers as predictors of death occurrence

We evaluated clinical characteristics and biomarkers to predict death occurrence during hospitalization. Age >75th percentile (>51.5 years) was associated with death occurrence (OR, 12.51 [1.98-153.7]; *P* = .016) (Supplementary Table 4). Dyslipidemia and initial hemorrhagic parenchymal lesion were associated with death occurrence (OR, 25.38 [3.11-524.2], *P* = .028; OR, 21.32 [2.63-439.2], *P* = .0045; respectively). No other clinical characteristics were associated with death occurrence. In unadjusted logistic regression, D_0_ hs-CRP level >75th percentile (>29.7 mg/L) was associated with odds of death (OR, 10.76 [1.55-140.4], *P* = .04). D_0_ D-dimer level >75th percentile (>2198 mg/L) was not associated with death occurrence (OR, 1.272 [0.20-15.95]; *P* = .99). The OR of death for D_5_ hs-CRP >75th percentile (>13.7 mg/L) was 13.08 (2.06-160.8) (*P* = .01), and for D_5_ D-dimer >75th percentile (>1060 mg/L), it was 14.63 (2.28-179.9) (*P* = .01).

#### 3.5.1 Evolution at 3 months

Imaging was evaluated at 3 months (Supplementary Table 3): 4 patients had worse imaging findings, 15 patients had no change, and 193 patients had improved imaging findings with partial (n = 112) or complete (n = 81) recanalization. The improvement was associated with a lower D_0_ NLR than that with no change (4.10 [2.54-5.76] vs 6.37 [5.02-9.57], respectively; *P* = .0246). No significant differences were observed for hs-CRP, D-dimer, or TG parameters. No laboratory marker predicted improvement, worsening, or stable modified Rankin Score.

## 4 Discussion

In our prospective study including 231 patients with CVST, we identified several biomarkers associated with clinical presentation characteristics and/or predictors of evolution. We confirmed, in a limited number of events, that hemorrhage and elevation of inflammation biomarkers, particularly hs-CRP and D-dimer, were associated with early death.

CVST is a rare thrombotic disorder caused by systemic or local imbalances in hemostasis [9]. Prognosis is usually good, with more than 80% of the patients making a complete recovery [5,6]. Despite the highly favorable prognosis, identification of patients with CVST having a possible unfavorable outcome can be challenging. For example, in a large cohort of 642 patients from the International Study on Cerebral Vein and Dural Sinus Thrombosis, clinical factors of unfavorable outcome have been identified: namely, male sex, age <37 years, coma, mental status disorder, intracranial hemorrhage on admission, thrombosis of the deep cerebral venous system, central nervous system infection, and cancer [5]. More recently, a practical clinical score was established to predict outcomes after CVST [20]. The clinical score associating parenchymal lesion size of >6 cm, bilateral Babinski signs, male sex, parenchymal hemorrhage, and level of consciousness are associated with unfavorable outcomes. However, no laboratory marker had been evaluated in these 2 studies. We confirmed that age >52 years and hemorrhagic parenchymal lesions were associated with unfavorable outcomes. We also found that dyslipidemia was associated with unfavorable outcomes; this abnormality has never been reported before. However, the confidence interval of the odds ratio is very wide, and this result deserves to be cautiously interpreted.

The association of inflammation biomarkers with diagnosis, severity, and poor prognosis of cerebrovascular events has already been reported, especially in patients with CVST [15,[20], [21], [22], [23], [24], [25]]. Recently, a multicenter prospective observational study was conducted in Portugal: the Pathophysiology of Venous Infarction – Prediction of Infarction and Recanalization in CVT (PRIORITy-CVT) study, including 62 patients, demonstrated that biomarkers associated with inflammation predicted poor prognosis in cerebral vein thrombosis (CVT) [15]. In this study, patients had more unfavorable outcomes than the FPCCVT cohort (21% vs 3% of deaths, respectively). Three biomarkers, namely NLR, CRP, and interleukin (IL)-6, were evaluated as predictors of evolution. As reported in our study, NRL was associated with initial parenchymal brain lesions [16]. A higher baseline NLR, CRP, and IL-6 levels were predictors of unfavorable functional outcome at 90 days [16,24]. We demonstrated in our cohort that baseline systemic inflammation biomarkers were also associated with poor outcomes during hospitalization. Finally, we reported a non-significant fibrinogen increase associated with parenchymal lesions and an increasing number of sinus veins with thrombosis, as well as a lower baseline NLR in patients with improvement in imaging findings at 3 months. Noteworthy, in the PRIORITy-CVT, higher NLR, CRP, and IL-6 levels at admission were not associated with the early evolution of brain lesions or early recanalization. In a recent study including 52 CVST, the hs-CRP level was significantly higher in patients with acute or subacute CVT than in patients with chronic CVT [25]. However, in our study, including a much higher number of patients, we did not find a significant difference.

In addition, we studied D-dimer levels. The D-dimer increase at diagnosis was associated with neurological deficit [22]. In our FPCCVT cohort, the initial D-dimer levels were associated with initial consciousness disturbance, early death occurrence, and the severity of parenchymal lesions. Together with hs-CRP, D-dimer measured at D_5_ predicted early death. The association between inflammatory biomarkers and clinical outcomes has been reported in different studies [16,26]. Interestingly, a previous study demonstrated that the D-dimer decrease between D_0_ and day 180 was associated with complete sinus recanalization in MRI findings at 6 months [21]. In a study evaluating fibrin clot properties, an increase in D-dimer was observed in patients who had developed recurrent CVST [27]. An altered fibrin clot and fibrinogen increase were observed in patients with recurrent CVST [28] and in patients with CVST-related pregnancy [29]. The combined activation of inflammatory and hemostatic responses after infection or tissue injury is a defense mechanism. This process is referred to as immunothrombosis: within this novel concept, inflammatory cells such as leukocytes and platelets directly drive the progression of venous thrombosis [30]. Under pathological conditions, an increase in tissue factor expression and increased levels of the plasminogen activation inhibitor 1, blocking fibrinolysis, amplify coagulation [31]. The elevation of D-dimer in our patients at baseline in parallel to that of other inflammation biomarkers shows that D-dimer is an acute phase reactant [32,33]. Once anticoagulant treatment had been initiated, the subsequent D-dimer decrease makes the interpretation of D-dimer more complex, although D-dimer measured on D_5_ still predicted early death.

Finally, in our cohort, TG parameters measured on D_0_, namely, ETP and velocity, were predictive of the presence of ischemic parenchymal lesions and a greater number of thrombosed sinuses. In the literature, to our knowledge, no study had previously evaluated TG as a predictor of CVST evolution. TG assay is a global coagulation assay that may be altered in hypercoagulable states [12,33] or different types of endothelial injury [[34], [35], [36]]. In our study, lower ETP_5pM_ was associated only with hemorrhagic parenchymal lesion development.

The strengths of our study were a prospective study design, with a prolonged follow-up, together with clinical and imaging evaluation at similar time points, including the evolution of parenchymal brain lesions and functional outcomes. However, our study has some limitations. Whereas inflammation markers and D-dimer are routinely performed, TG assay is performed only in some laboratories, and the calibrated automated thrombogram system is poorly standardized. We had a few severe clinical presentations. However, Coutinho et al. [37] showed an improvement over time in initial CVT presentations due to improved diagnostic methods. We have demonstrated that the FPCCVT cohort had similar clinical, radiological, biological, and etiological characteristics to the historical International Study on Cerebral Vein and Dural Sinus Thrombosis cohort, suggesting the generalizability of our results. Contrarily, clinical prediction of CVST outcomes is likely complicated, involving several patient characteristics, especially underlying comorbid conditions. Also, we could not assess race or ethnicity as a social determinant related to our findings for regulatory reasons. The power was limited in that we only had 5 deaths and therefore could not perform age-adjusted or multivariable analysis.

In conclusion, we reported here that 2 widely available biomarkers, namely hs-CRP and D-dimer, could help predict early death in CVST in addition to patient characteristics. These results need to be validated in external cohorts.

## References

[1] Payne A.B., Adamski A., Abe K., Reyes N.L., Richardson L.C., Hooper W.C. (2022). Epidemiology of cerebral venous sinus thrombosis and cerebral venous sinus thrombosis with thrombocytopenia in the United States, 2018 and 2019. Res Pract Thromb Haemost.

[2] Kristoffersen E.S., Harper C.E., Vetvik K.G., Zarnovicky S., Hansen J.M., Faiz K.W. (2020). Incidence and mortality of cerebral venous thrombosis in a Norwegian population. Stroke.

[3] Coutinho J.M., Zuurbier S.M., Aramideh M., Stam J. (2012). The incidence of cerebral venous thrombosis: a cross-sectional study. Stroke.

[4] Stam J. (2005). Thrombosis of the cerebral veins and sinuses. N Engl J Med.

[5] Ferro J.M., Canhão P., Stam J., Bousser M.G., Barinagarrementeria F. (2004). Prognosis of cerebral vein and dural sinus thrombosis: results of the International Study on Cerebral Vein and Dural Sinus Thrombosis (ISCVT). Stroke.

[6] Bousser M.G., Ferro J.M. (2007). Cerebral venous thrombosis: an update. Lancet Neurol.

[7] Alons I.M., Jellema K., Wermer M.J., Algra A. (2015). D-dimer for the exclusion of cerebral venous thrombosis: a meta-analysis of low-risk patients with isolated headache. BMC Neurol.

[8] Dentali F., Squizzato A., Marchesi C., Bonzini M., Ferro J.M., Ageno W. (2012). D-dimer testing in the diagnosis of cerebral vein thrombosis: a systematic review and a meta-analysis of the literature. J Thromb Haemost.

[9] Silvis S.M., de Sousa D.A., Ferro J.M., Coutinho J.M. (2017). Cerebral venous thrombosis. Nat Rev Neurol.

[10] Kasonga F., Feugray G., Chamouni P., Barbay V., Fresel M., Chretien M.H. (2021). Evaluation of thrombin generation assay in factor XI deficiency. Clin Chim Acta.

[11] Feugray G., Kasonga F., Chamouni P., Barbay V., Fresel M., Chretien M.H. (2021). Factor XII deficiency evaluated by thrombin generation assay. Clin Biochem.

[12] Billoir P., Duflot T., Fresel M., Chrétien M.H., Barbay V., Le Cam Duchez V. (2019). Thrombin generation profile in non-thrombotic factor V Leiden carriers. J Thromb Thrombolysis.

[13] Billoir P., Le Cam Duchez V., Miranda S., Richard V., Benhamou Y. (2021). Thrombin generation assay in autoimmune disease. Rev Med Intern.

[14] Billoir P., Miranda S., Levesque H., Benhamou Y., Le Cam Duchez V. (2021). Hypercoagulability evaluation in antiphospholipid syndrome without anticoagulation treatment with thrombin generation assay: a preliminary study. J Clin Med.

[15] Sonnevi K., Tchaikovski S.N., Holmström M., Rosing J., Bremme K., Lärfars G. (2011). Thrombin generation and activated protein C resistance in the absence of factor V Leiden correlates with the risk of recurrent venous thromboembolism in women aged 18-65 years. Thromb Haemost.

[16] Aguiar de Sousa D., Pereira-Santos M.C., Serra-Caetano A., Lucas Neto L., Sousa A.L., Gabriel D. (2021). Blood biomarkers associated with inflammation predict poor prognosis in cerebral venous thrombosis: a multicenter prospective observational study. Eur J Neurol.

[17] Kaya H., Ertaş F., İslamoğlu Y., Kaya Z., Atılgan Z.A., Çil H. (2014). Association between neutrophil to lymphocyte ratio and severity of coronary artery disease. Clin Appl Thromb Hemost.

[18] Triquenot Bagan A., Crassard I., Drouet L., Barbieux-Guillot M., Marlu R., Robinet-Borgomino E. (2021). Cerebral venous thrombosis: clinical, radiological, biological, and etiological characteristics of a French prospective cohort (FPCCVT)—comparison with ISCVT cohort. Front Neurol.

[19] Bousser M., Russel R.R., Warlow C.P., Van Gijn J. (1997). Major problems in neurology.

[20] Barboza M.A., Chiquete E., Arauz A., Merlos-Benitez M., Quiroz-Compeán A., Barinagarrementería F. (2018). A practical score for prediction of outcome after cerebral venous thrombosis. Front Neurol.

[21] Meng R., Wang X., Hussain M., Dornbos D., Meng L., Liu Y. (2014). Evaluation of plasma D-dimer plus fibrinogen in predicting acute CVST. Int J Stroke.

[22] Juli C., Amalia L., Gamayani U., Atik N. (2020). D-dimer level associates with the incidence of focal neurological deficits in cerebral venous thrombosis patients. J Blood Med.

[23] Akboga Y.E., Bektas H., Anlar O. (2017). Usefulness of platelet to lymphocyte and neutrophil to lymphocyte ratios in predicting the presence of cerebral venous sinus thrombosis and in-hospital major adverse cerebral events. J Neurol Sci.

[24] Wang L., Duan J., Bian T., Meng R., Wu L., Zhang Z. (2018). Inflammation is correlated with severity and outcome of cerebral venous thrombosis. J Neuroinflammation.

[25] Duan J., Leng X., Han Z., Cai Y., Wang C., Rajah G. (2021). Identifying biomarkers associated with venous infarction in acute/subacute cerebral venous thrombosis. Aging Dis.

[26] de Sousa D.A., Pereira-Santos M.C., Serra-Caetano A., Neto L.L., Sousa A.L., Gabriel D. (2021). Matrix metalloproteinase-9 levels are associated with brain lesion and persistent venous occlusion in patients with cerebral venous thrombosis. Thromb Haemost.

[27] Skuza A.A., Polak M., Undas A. (2019). Elevated lipoprotein(a) as a new risk factor of cerebral venous sinus thrombosis: association with fibrin clot properties. J Thromb Thrombolysis.

[28] Siudut J., Świat M., Undas A. (2015). Altered fibrin clot properties in patients with cerebral venous sinus thrombosis: association with the risk of recurrence. Stroke.

[29] Liang Z.W., Gao W.L., Feng L.M. (2017). Clinical characteristics and prognosis of cerebral venous thrombosis in Chinese women during pregnancy and puerperium. Sci Rep.

[30] Heestermans M., Poenou G., Duchez A.C., Hamzeh-Cognasse H., Bertoletti L., Cognasse F. (2022). Immunothrombosis and the role of platelets in venous thromboembolic diseases. Int J Mol Sci.

[31] Jackson S.P., Darbousset R., Schoenwaelder S.M. (2019). Thromboinflammation: challenges of therapeutically targeting coagulation and other host defense mechanisms. Blood.

[32] Prucha M., Bellingan G., Zazula R. (2015). Sepsis biomarkers. Clin Chim Acta.

[33] Billoir P., Alexandre K., Duflot T., Roger M., Miranda S., Goria O. (2021). Investigation of coagulation biomarkers to assess clinical deterioration in SARS-CoV-2 infection. Front Med.

[34] Billoir P., Miranda S., Damian L., Richard V., Benhamou Y., Duchez V.L. (2018). Development of a thrombin generation test in cultured endothelial cells: evaluation of the prothrombotic effects of antiphospholipid antibodies. Thromb Res.

[35] Billoir P., Blandinières A., Gendron N., Chocron R., Gunther S., Philippe A. (2021). Endothelial colony-forming cells from idiopathic pulmonary fibrosis patients have a high procoagulant potential. Stem Cell Rev Rep.

[36] Détriché G., Gendron N., Philippe A., Gruest M., Billoir P., Rossi E. (2022). Gonadotropins as novel active partners in vascular diseases: insight from angiogenic properties and thrombotic potential of endothelial colony-forming cells. J Thromb Haemost.

[37] Coutinho J.M., Zuurbier S.M., Stam J. (2014). Declining mortality in cerebral venous thrombosis: a systematic review. Stroke.

