# Supplementary material for: C-reactive protein and D-dimer in cerebral vein thrombosis: Relation to clinical and imaging characteristics as well as outcomes in a French cohort study
Source: Res Pract Thromb Haemost. 2023 Mar 28;7(3):100130. doi: 10.1016/j.rpth.2023.100130 (PMC10149398; doi:10.1016/j.rpth.2023.100130)
Supplement: Supplementary Table 1 [file mmc1.docx]

| **Parameters** | **All**  **(n=231)** |
| --- | --- |
| Haemoglobin (g/dL) | 13.7 [2.4-21.5] |
| Hs-CRP (mg/L) | 10.6 [0.2-159] |
| Hs-CRP>5mg/dL | 151 (65.4%) |
| NLR | 3.75 [0.18-29.2] |
| D-dimer (µg/L) | 1050 [270-26700] |
| D-dimer>500 µg/L | 191 (82.7%) |
| Fibrinogen (g/L) | 4.0 [0.51-7.6] |
| Lagtime_1pM_ (min) | 7.92 [3.33-21.3] |
| TTP_1pM_ (min) | 11.81 [5.42-26.64] |
| ETP_1pM_ (nM•min) | 1338 [109-3134] |
| Peak_1pM_ (nM) | 205 [6.88-622] |
| Velocity_1pM_ (nM/min) | 54.3 [0.4-306.6] |
| Lagtime_5pM_ (min) | 4.17 [2.29-11.8] |
| TTP_5pM_ (min) | 7.21 [3.96-25.3] |
| ETP_5pM_ (nM•min) | 1657 [348-3047] |
| Peak_5pM_ (nM) | 286 [6.9-544] |
| Velocity_5pM_ (nM/min) | 94.1 [0.40-298] |

**Supplemental Table 1. Laboratory markers on D_0_.** Results are expressed as median (Min-Max). TTP: time to peak. ETP: endogenous thrombin potential. NLR: neutrophil to lymphocyte ratio. HS-CRP: high sensitivity C-reactive protein.
