# Supplementary material for: C-reactive protein and D-dimer in cerebral vein thrombosis: Relation to clinical and imaging characteristics as well as outcomes in a French cohort study
Source: Res Pract Thromb Haemost. 2023 Mar 28;7(3):100130. doi: 10.1016/j.rpth.2023.100130 (PMC10149398; doi:10.1016/j.rpth.2023.100130)
Supplement: Supplementary Table 2 [file mmc2.doc]

| Parameters | N | hs-CRP (mg/l) | NLR | D-dimer (µg/L) | Fibrinogen  (g/L) | Lagtime_1pM_ (min) | TTP_1pM_ (min) | ETP_1pM_ (nM•min) | Peak_1pM_ (nM) | Velocity_1pM_ (nM/min) | Lagtime_5pM_ (min) | TTP_5pM_ (min) | ETP_5pM_ (nM•min) | Peak_5pM_ (nM) | Velocity_5pM_ (nM/min) |
| --- | --- | --- | --- | --- | --- | --- | --- | --- | --- | --- | --- | --- | --- | --- | --- |
| **NIHSS variation between D_0_ to D_5_** |  | | | | | | | | | | | | | | |
| *Stable* | 159 | **9.6 [2.8-23.4]** | **3.5 [2.1-5.9]** | 880 [500-1640] | 4.0 [3.4-4.7] | 7.7 [6.0-9.2] | 11.8 [10.0-13.5] | 1269 [933-1736] | 190 [110-274] | **49.8 [23.0-83.1]** | 4.0 [3.3-4.8] | 7.4 [6.0-8.2] | 1657 [1368-2053] | 290 [214-378] | 92.7 [56.5-142.1] |
| *Improvement* | 59 | **13.4 [5.7-31.4]** | **3.9 [2.1-6.5]** | 1340 [856-2365] | 4.1 [3.5-4.8] | 7.9 [6.5-9.1] | 11.8 [10.0-13.6] | 1366 [1088-1804] | 21 [129-303] | **53.9 [26.9-110.6]** | 4.2 [3.5-5.4] | 7.5 [6.5-9.1] | 1635 [1422-2110] | 277 [215-379] | 92.0 [55.5-147.1] |
| *Worsening* | 11 | **32.7 [18.6-105.1]** | **6.9 [4.7-12.7]** | 2430 [1230-4315] | 4.3 [3.5-4.5] | 7.9 [6.0-9.9] | 10.8 [8.6-13.2] | 1521 [1325-1950] | 248 [212-377] | **74.4 [63.6-157.8]** | 4.3 [3.9-5.1] | 7.1 [6.3-8.4] | 1863 [1559-2104] | 364 [271-436] | 135.6 [81.2-181.2] |
| P value |  | ***P=0.003*** | ***P=0.0041*** |  |  |  |  |  |  | ***P=0.016*** |  |  |  |  |  |
| **Rankin variation between D_5_ to M_3_** |  | | | | | | | | | | | | | | |
| *Stable* | 95 | 10.3 [3.0-22.7] | 3.9 [2.3-5.9] | 1040 [623-2248] | 3.8 [3.4-4.6] | 7.8 [6.3-9.2] | 11.8 [10.0-13.5] | 1360 [1004-1802] | 209 [118-288] | 55.5 [25.0-85.4] | 3.9 [3.3-4.6] | 7.2 [6.0-8.0] | 1761 [1393-2223] | 302 [323-385] | 106.7 [65.3-145.9] |
| *Improvement* | 70 | 10.0 [4.0-31.4] | 4.0 [2.4-6.7] | 910 [493-2175] | 4.1 [3.4-4.9] | 7.7 [6.0-8.8] | 11.7 [9.1-13.2] | 1317 [942-1758] | 185 [119-289] | 49.2 [24.3-98.2] | 4.1 [3.5-4.8] | 7.1 [6.3-8.5] | 1649 [1365-2048] | 283 [194-388] | 96.9 [54.3-145.6] |
| *Worsening* | 47 | 11.6 [4.2-30.8] | 2.9 [1.7-4.8] | 1130 [550-1660] | 4.1 [3.6-5.0] | 7.3 [5.8-8.4] | 10.9 [9.4-13.2] | 1245 [1080-1679 | 220 [98-281] | 63.2 [20.2-108.0] | 4.2 [3.3-6.1] | 7.5 [6.5-9.6] | 1465 [1145-1864] | 265 [191-352] | 82.9 50.8-130.9] |
| **Outcome** |  |  |  |  |  |  |  |  |  |  |  |  |  |  |  |
| *No death* | 228 | **10.6 [3.6-29.1]** | 3.8 {2.2-6.1] | **965 [550-2123]** | 4.0 [3.4-4.7] | 7.7 [6.0-8.9] | 11.7 [10.0-13.3] | 1343 [1004-1751] | 207 [116-281] | 54.1 [24.8-86.2] | 4.1 [3.3-4.8] | 7.2 [6.3-8.3] | 1661 [1384-2041] | 291 [215-374] | 93.2 [56.9-142.1] |
| *Death* | 5 | **71.2 [19.0-110.8]** | 8.0 [0.18-11.5] | **2405 [2085-10250]** | 4.3 [3.5-6.4] | 9.8 [8.3-10.2] | 12.9 [10.8-14.2] | 1309 [1130-1490] | 220 [168-351] | 74.0 [43.6-149.6] | 5.4 [4.1-6.9] | 8.4[6.9-11] | 1518 [1399-1963] | 250 [213-387] | 78.7 [57.9-151.0] |
| *P value* |  | ***P=0.017*** |  | ***P=0.020*** |  |  |  |  |  |  |  |  |  |  |  |
| **Imaging evolution at 3 months** |  | | | | | | | | | | | | | | |
| *Improvement* | 66 | 14.3 [5.6-31.3] | **4.1 [2.5-5.8]** | 1105 [645-2278] | 4.3 [3.6-4.9] | 7.5 [6.5-8.8] | 11.7 [10.1-13.1] | 1454 [1121-1857] | 221 [146-292] | 64.5 [32.0-90.3] | 4.1 [3.5-4.9] | 7.1 [6.5-8.1] | 1766 [1437-2243] | 326 [223-421] | 150.1 [60.7-153.2] |
| *Stable* | 13 | 16.6 [3.4-126.8] | **6.4 [5.0-9.6]** | 2350 [885-3173] | 4.4 [3.7-4.9] | 8.3 [7.0-9.2] | 12.4 [10.2-15.1] | 1334 [478-1881] | 235 [64-331] | 65.3 [11.9-119.4] | 4.5 [3.8-6.7] | 7.7 [6.1-10.6] | 1594 [779-2173] | 327 [108-396] | 106 .3 [29.0-160.3] |
| *P value* |  |  |  | ***P=0.016*** |  |  |  |  |  |  |  |  |  |  |  |

**Supplemental Table 2. Laboratory markers on D_0_ associated with clinical and imaging evolution.** Results are expressed as median (interquartile range). Results in bold indicate a significant difference. TTP: time to peak. ETP: endogenous thrombin potential. NLR: neutrophil to lymphocyte ratio. Hs-CRP: high sensitivity C-reactive protein. ICHT: intracranial hypertension. Statistical differences are in bold, with a P value below. ^c^ comparison between focal syndrome and ICHT,  ^b^ comparison between focal syndrome and diffuse encephalopathy. N was the number of test available in patient.
