# Supplementary material for: C-reactive protein and D-dimer in cerebral vein thrombosis: Relation to clinical and imaging characteristics as well as outcomes in a French cohort study
Source: Res Pract Thromb Haemost. 2023 Mar 28;7(3):100130. doi: 10.1016/j.rpth.2023.100130 (PMC10149398; doi:10.1016/j.rpth.2023.100130)
Supplement: Supplementary Table 3 [file mmc3.docx]

| Parameters | Age | Sex | BMI | NLR | D-dimer | Fibrinogen | Lagtime_1pM_ | TTP_1pM_ | ETP_1pM_ | Peak_1pM_ | Velocity_1pM_ | Lagtime_5pM_ | TTP_5pM_ | ETP_5pM_ | Peak_5pM_ | Velocity _5pM_ |
| --- | --- | --- | --- | --- | --- | --- | --- | --- | --- | --- | --- | --- | --- | --- | --- | --- |
| *Hs-CRP* |  |  |  |  |  |  |  |  |  |  |  |  |  |  |  |  |
| p | 0.15 | <0.001 | 0.93 | <0.001 | <0.001 | <0.001 | 0.52 | 0.29 | <0.001 | 0.0032 | 0.001 | 0.046 | 0.44 | 0.087 | 0.038 | 0.0038 |
| R^2^ | 0.009 | 0.0625 | 4.2 10^-5^ | 0.097 | 0.089 | 0.13 | 0.0027 | 0.0070 | 0.071 | 0.053 | 0.061 | 0.025 | 0.0037 | 0.018 | 0.027 | 0.050 |
| D-dimer |  |  |  |  |  |  |  |  |  |  |  |  |  |  |  |  |
| P | 0.40 | 0.21 | 0.14 | 0.01 | NA | 0.053 | 0.43 | 0.23 | 0.032 | 0.1 | 0.13 | 0.60 | 0.79 | 0.19 | 0.27 | 0.26 |
| R^2^ | 0.0037 | 0.008 | 0.011 | 0.034 | NA | 0.022 | 0.004 | 0.009 | 0.030 | 0.018 | 0.015 | 0.0018 | 0.0004 | 0.011 | 0.008 | 0.008 |

**Supplemental Table 3. Correlation of D_0_ hs-CRP and D-dimer, with others clinical and biological characteristic’s.** TTP: time to peak. ETP: endogenous thrombin potential. NLR: neutrophil to lymphocyte ratio. Hs-CRP: high sensitivity C-reactive protein
