# Supplementary material for: C-reactive protein and D-dimer in cerebral vein thrombosis: Relation to clinical and imaging characteristics as well as outcomes in a French cohort study
Source: Res Pract Thromb Haemost. 2023 Mar 28;7(3):100130. doi: 10.1016/j.rpth.2023.100130 (PMC10149398; doi:10.1016/j.rpth.2023.100130)
Supplement: Supplementary Table 4 [file mmc4.doc]

| Patient characteristics | OR (95% CI) | P-value |
| --- | --- | --- |
| Age>51.5 years | 12.51 (1.98-153.7) | 0.0156 |
| Sex (female) | 0.45 (0.057-3.85) | 0.44 |
| BMI | 1.01 (0.79-1.14) | 0.8994 |
| Dyslipidemia | 25.38 (3.11-524.2) | 0.0028 |
| Tabacco | 3.16 (0.14-33.95) | 0.39 |
| Diabete | 7.23 (0.34-62.59) | 0.099 |
| Initial consciousness disturbance | 5.88 (0.69-50.37) | 0.099 |
| Hemorrhagic parenchymal lesion | 21.32 (2.63-439.2) | 0.0045 |

Supplemental table 4. Association factors with death.
